# Reply to Papini, A. Comment on “Nekkaa et al. *Rhamnus alaternus* Plant: Extraction of Bioactive Fractions and Evaluation of Their Pharmacological and Phytochemical Properties. *Antioxidants* 2021, *10*, 300”

**DOI:** 10.3390/antiox12122114

**Published:** 2023-12-14

**Authors:** Amine Nekkaa, Akila Benaissa, Fabrice Mutelet, Laetitia Canabady-Rochelle

**Affiliations:** 1 Process Engineering Laboratory for Sustainable Development and Health Products, Department of Process Engineering, National Polytechnic School of Constantine—Malek Bennabi, Constantine 25000, Algeria; 2 Laboratoire Réactions et Génie des Procédés, CNRS, Université de Lorraine, F-54000 Nancy, France; 3 Laboratory of Process Engineering for the Environment (LIPE), Department of Pharmaceutical Engineering, Faculty of Process Engineering, Salah Boubnider University, Constantine 3, Constantine 25000, Algeria

We appreciate the commentary by Alessio Papini [1] regarding our published article [2]. The taxonomy of species-rich genera often represents a challenge for scientists because reaching a sound classification requires extensive sampling. Furthermore, *Rhamnus alaternus* is an example of a species with a challenging taxonomic history, as is also the case for other genera of *Rhamnaceae*. In Section 2.2 (Botanica Description), the authors agreed to change the passage in question as follows: “*R. alaternus* belongs to the *Magnoliophyta* division, the *Magnoliopsida* class, the *Rhamnales* order, the *Rhamnaceae* family, the *Rhamnus* Genus and the *Rhamnus alaternus* species”.

The second point is the intraspecific treatment of *Rhamnus alaternus* by Nekkaa et al. [2]. We would like to thank Alessio Papini for his correction, and our intention was to mention these varieties. The authors would like to change this sentence according to the proposition of Alessio Papini: “In addition, this plant has several varieties including *R*. *a*. var *angustifolia* DC, *R. a*. var *balearica* DC, *R. a*. var *hispanica* DC, and *R. a*. var *vulgaris* DC”.

We now address the following pertinent question asked by Alessio Papini: how much does taxonomic inaccuracy affect the results about the pharmaceutical properties of plants? As a pharmaceutical and food-process-engineering team, we are working on the optimization of the green extraction of bioactive compounds from different parts of *Rhamnus alaternus* plants from Algeria. Before our experimental study was conducted, the botanical identification was overseen in collaboration with botanists and experts in phytochemistry from the laboratory of pharmacognosy, the Pharmacy department, University of Constantine 3 (Algeria), to ensure that we worked on *Rhamnus alaternus* plants specifically [3].

To respond to the question raised by Alessio Papini, pharmaceutical and food-process-engineering teams like ours must work in close collaboration with taxonomists and experts in phytochemistry to avoid taxonomic inaccuracy. Moreover, such collaboration would enable us to explore and understand how systematic biodiversity might influence the biodiversity of secondary metabolites in plants. Thus, we fully agree with the author of the commentary and look forward to hearing more about this promising topic in the near future.
